# Traditional therapeutic practices in the Himalayan heartland of Kinnaur, Himachal Pradesh, India - A qualitative exploration

**DOI:** 10.1016/j.jaim.2025.101262

**Published:** 2025-11-10

**Authors:** Abhishek Sharma, Qutsia Tabasum

**Affiliations:** a Department of Anthropology, University of Ladakh, UT Ladakh, 194105, India; b Department of Forensic Science, University of Ladakh, UT Ladakh, 194101, India

**Keywords:** Traditional medicine system, Traditional healers, Holistic treatment, Socio-cultural aspects, Medicinal plants

## Abstract

**Background:**

Traditional medicine systems have been essential components of health systems in rural areas, and areas with rich cultures and geographical isolation such as district Kinnaur. Derived from centuries of practice, these systems understand health and illness in terms of physical, mental, and socio-cultural well-being.

**Objectives:**

This study documents the structural and functional aspects of the traditional medicine system prevalent in district Kinnaur, Himachal Pradesh, India, with a focus on understanding how traditional healers understand health, how they diagnose illness, and how they treat illnesses using traditional methods.

**Material and methods:**

The present research was conducted in district Kinnaur, in the western Himalayas of Himachal Pradesh, India. This study is an exploratory qualitative research utilizing a thematic analysis approach (Braun and Clarke, 2006) to explore the traditional medicine system. The researcher collected data from all practicing traditional healers using an in-depth interview guide for the sessions.

**Results:**

The traditional medicine system of district Kinnaur provides a holistic assessment of health and treatment of illness, incorporating all the aspects of the individual (physical, psychological, and socio-cultural). Structural and functional aspects of the traditional medicine system are documented under 6 major themes and sub-themes, which are 1) Concept of health, 2) Aetiology of Illness, 3) Diagnostic techniques, 4) Treatment methods, 5) Training and Learning process, 6) Production of medicine.

**Conclusion:**

Traditional healers of Kinnaur have long classified, examined, and applied natural resource knowledge to develop therapeutic remedies. Understanding sociocultural patterns will aid policymakers in framing policies that would help sustain the age-old traditional knowledge of medicine, and it will also inform and sensitize healthcare providers to the beliefs and practices of their patients. In-depth interdisciplinary studies on the area's natural resources should be undertaken to understand active compounds of herbal plants used by traditional healers, which can validate traditional medicine through biomedical research.

## 1 Introduction

Every society grapples with fundamental health-related challenges, and to address these challenges, various societies have created different healthcare systems, also known as medical systems [1]. In some African and Asian nations, up to 80 % of the population relies on traditional medicine for their primary healthcare needs [2]. In India, many people depend on traditional medicine, and there are different traditional medicine systems in other parts of the country [3]. Traditional medicine is a system of knowledge that has developed through generations, long before the advent of modern medicine [4]. The WHO defines traditional medicine as “the health practices, approaches, knowledge, and beliefs incorporating plant, animal and mineral-based medicines, spiritual therapies, manual techniques, and exercises, applied singularly or in combination to treat, diagnose and prevent illnesses or maintain well-being” [5].

Prevalent in many simple societies, the traditional medicine system is a specialized healthcare service where designated individuals referred to as traditional healers or practitioners undergo extensive training to provide healing services to a group. Traditional healthcare practitioners have remained essential to the healthcare of their people primarily because they can match the expectations of the people in their communities through their understanding of language, cultural values, and behavioural patterns [6,7,8]. The use of traditional medicinal plants by the people of Kinnaur has been documented by Kshtriya & Aniket [9].

Given concerns about the possible loss of culture and values, including traditional medicine systems, which are an integral part of the culture, there is an urgent need to understand the structural and functional aspects of traditional medicine for its scientific validation [10].

Various scholars have conducted many ethnobotanical studies to understand the use of medicinal plants by different tribal and non-tribal communities in India. However, the knowledge of structural and functional aspects of traditional medicine systems is missing in the literature. The present research is a part of PhD research conducted in District Kinnaur, Himachal Pradesh, India. It explores the structural and functional dimensions of the traditional medicine system prevalent in the tribal district of Kinnaur, Himachal Pradesh (India). Structural dimensions focuses on transmission of knowledge, institutional/non-institutional setup, and community recognition, while functional dimensions focuses on diagnostic approaches, treatment methods, and local of health and illness. The research was guided by the following research questions.

• How do traditional healers perceive and conceptualize health and illness?
• What are the diagnostic and treatment techniques employed by traditional healers of Kinnaur?
• How is traditional medicinal knowledge transmitted to the next generation, and what roles do family and community play in this process?

## 2 Methodology

Research team and reflexivity: Our research team consisted of two researchers (AS and QT), one senior anthropologist, and another junior anthropologist, each bringing a wealth of expertise to the study. The team leader (AS) with over 4 years of experience in qualitative research, held a Master's degree and was pursuing a PhD at the time of data collection. The junior researchers possessed a bachelor's degree during data collection. The research team comprised a mixture of male (n = 1) and female (n = 1) members. Before the data collection, the junior researcher underwent comprehensive training for five months. Before beginning in-depth interviews (IDIs) with the traditional healers, the researchers spent time in the field introducing the study's research questions and establishing rapport with participants.

**Study Design:** It employs a qualitative research approach using thematic analysis [11] to explore the structural and functional dimensions of the traditional medicine system. This approach focuses on diagnostic and treatment methods utilized by traditional healers.

## 3 Study setting

The research has been conducted in the district Kinnaur of Himachal Pradesh, India. The state of Himachal Pradesh, is nestled in the western Himalayas, surrounded by the state of Jammu and Kashmir in the north, Punjab in the west, and Tibet (international border) in the east. Data collection was conducted in selected villages of all three development blocks of Kinnaur district, Himachal Pradesh. For administrative purposes, the district is divided into three Development Blocks. Block 1 (Nichar) is Lower Kinnaur, Block 2 (Kalpa) Middle Kinnaur, and Block 3 (Pooh), which is situated at a higher altitude, as Upper Kinnaur. Villages such as Tapri, Ralli (Nichar Block), Kalpa, Roghi (Kalpa Block), and Nako, Chango (Pooh Block) were among the selected sites for data collection. The three blocks are distinct in their geography and socio-culture. District Kinnaur was notified as a Scheduled Area due to its challenging topography and unique cultural background, and all its residents were given rights and privileges as Scheduled Tribes under the Constitution of India. The region's native tribal population is notified as ‘Kinnaura’ officially. The district has a predominantly tribal population, with Scheduled Tribes constituting over 95 % of the total population [12].

## 4 Sample size and data collection

A qualitative data collection method of in-depth interviews was used to collect data. Using an in-depth interview guide prepared by the researchers, the data were collected from all the practicing traditional healers of Kinanur. The in-depth interview guide was semi-structured, comprising open-ended questions organized around key themes such as health beliefs, diagnostic practices, treatment processes, and knowledge transmission. Data collection occurred over a six-month period, from April to September 2018. Men and women aged 18 years and above were eligible for the research. Purposive sampling was used to select the respondents. Traditional healers were contacted personally and via mobile phones to inform them about the purpose of the research. In District Kinnaur seventeen traditional medicine healers were identified and contacted for in-depth interviews. Because of the small and specified number of traditional healers in the area, the study used a complete population sampling strategy, which included all seventeen healers who were approached for the in-depth interview. Data was collected at the homes of these traditional healers, from where most of them practice healing. Both researchers were present at the time of data collection along with the participant. Each interview lasted approximately 50–60 min. Interview notes were taken by one of the researchers, and an audio recording was made to collect data. There were no repeat interviews as data was comprehensively collected during initial interaction. Data saturation was not sought as the sample size was small, and we wanted to extract maximum information. Before conducting interviews with the participants, prior informed consent was sought from all the participants of the research. Details of the traditional healers are provided in Table 1.

**Table 1:** Demographic Profile of Traditional healers.

Demographic Variables	Gender		Age group			Religion	
Number of Traditional Healers	Male	Female	50–60	61–70	71–80	Hinduism	Buddhism
	16	1	12	3	2	6	11

## 5 Data analysis

To facilitate analysis, the interviews were transcribed manually into written documents, and the accuracy of these transcriptions was reviewed. Transcripts were not returned to participants for comments or corrections to avoid potential biases in responses. Instead, both researchers rigorously checked the accuracy of transcriptions prepared to ensure data reliability. Data was coded and recoded for common phrases and emerging patterns by both researchers. The coding tree was developed during the initial stages of data analysis, however it is not presented in this manuscript. Data were analyzed manually through inductive coding to derive themes directly from the participants' responses, employing the thematic analysis [11] approach. Similar codes falling under broad themes were pooled and organized, overlapping themes were evaluated, and final themes and sub-themes were identified. Typical quotes are documented to validate the themes and sub-themes. The identifiers for the quotes include the age and gender of traditional healers. The compiled themes were not shared with participants for feedback to minimize the risk of biasing their perspective.

## 6 Findings

The research documents the traditional medicine system in District Kinnaur, which has served as the foundation of healthcare in Kinnaur for generations. Structural and functional aspects of the traditional medicine system are documented under 6 major themes, including various subthemes. Major themes are: 1) Concept of health, 2) Aetiology of illness, 3) Diagnostic techniques, 4) Treatment methods, 5) Training and Learning process, 6) Production of medicine.

### 6.1 Traditional medicine system of Kinnaur

The traditional medicine system of Kinnaur is primarily based on the regional flora, and herbal medicine is the predominant type of therapeutic measure. The traditional medicine system in Kinnaur is an age-old informal setup catering to the local population's healthcare needs. Local traditional healers practice independently, without any organizational structures. The decision to seek healthcare is heavily influenced by socio-cultural factors in Kinnaur, making the traditional medicine system crucial to the region's healthcare culture. Traditional healers are locally known as ‘*Amchi*’ derived from the Mongolian language meaning ‘Superior to all’, are trusted to diagnose illness and administer treatment. They play a vital role in promoting overall health among the residents of Kinnaur. The following themes and sub-themes explore the structural and functional aspects of the traditional medicine system.

### 6.2 Theme 1. Concept of health

Traditional healers believe that the balance of body elements, the relationship between the body and environment, and food intake are the main principles around which an individual's health revolves. Health is viewed as a function of the human body, emphasizing its holistic nature rather than a mere physiological aspect.

**Sub Theme 1.1. Balance (Santulan) of three** *Doshas:* Health is seen as the balance between the three humors of the body. It is believed that health is maintained by three *Doshas*: vata, pitta, and kapha in the local dialect known as *vayu, thema,* and *pakhad*. These humors are basics for categorizing the human physique into three divisions similar to the concept of body composition in anthropometry, i.e., ectomorph, mesomorph, and endomorph. Humors are the body forces that determine the growth and development of the body.

*Vata* is referred to as wind inside the body. It governs the major functions of all the body organs, from blood flow to thinking power. Its movement is present throughout the human body, traveling through all systems and fulfilling its primary function of enabling the mobility of all organs. It also balances the other two humors, bile and phlegm.

Pitta represents the fire within the body and is a fluid secreted by the liver. It plays a crucial role in digestion, appetite, and metabolism, and is characterized by the body's heat. Additionally, bile helps in the elimination of waste products, including feces and urine. It is produced in the liver, stored in the gallbladder, and contributes to the digestive processes in the stomach, acting as the body's digestive fire.

*Kapha* is a watery element of the body that maintains nourishment supplies and proper lubrication of different organs. It controls our sensory organs and is responsible for overweight. The following narratives from the field allow us to understand how traditional healers construct the meaning of health.

“Every individual has different levels of three humors which shape their physique and balance between these three is necessary for healthy life” (Aged 59, Male)

**Sub Theme 1.2. Health as a function of five elements*:*** Traditional healers believe that the balance of five body elements should also be attained to have a healthy life. The concept that the human body is made up of 5 elements: Earth or *Prithvi* (Bones and Muscles), Water (*Jal*) or Blood, Air or *Vayu* (Breath), Fire or *Agni* (Heat), and Space or *Akash* (Emptiness) is prevalent among the traditional healers and health is seen as a function of these elements.

**Sub theme 1.3. Health as a function of digestive system (liver):** Good health is also understood in terms of the functioning of one's digestive system (liver) and it is of paramount importance that the liver remains healthy to keep rest of the organs and whole body in shape. Furthermore, to achieve this goal, one must have a balanced diet. It is the key to proper functioning of the physical body and psychological functions.

*“A healthy person is recognized by proper functioning of his digestive system and liver. If one takes a balanced diet one shall remain healthy since the digestive system will be fine*.” (Aged 52, Female)

Health primarily depends on one's diet. Food is also recognized as a preventive measure against various illnesses.

**Sub theme 1.4. Health as a function of mind and body***:* Traditional healers believe that a healthy person can work effectively and maintain a stable mind. They define physical work as the labor done primarily on farms, while a stable mind refers to an individual's behavior being in harmony with societal norms.

*“Until body and mind function properly, the individual remains healthy”* (Aged 54, Male)

### 6.3 Theme 2. Aetiology of illness

Illness is referred to as “*Bimari*” by the traditional healers and community members. One is considered ill when he/she cannot perform daily activities, which in an agricultural setup is a purely physical workout in the fields. Illness relates not only to one's biological aspect but to various other aspects of the human body and environment. As the concept of health among the healers of Kinnaur is based on the balance of different fluids and elements of the body, the concept of illness is based on the imbalance known as “*asantulan*”. However, it is believed that some illnesses, such as cancer, are purely based on the new lifestyle, instead of the concept of “imbalance”. Healers perceive illness purely in the social and cultural context as an aberration from the normal functioning of the body.

“One who cannot work in fields is ill” (Aged 62, Male)

**Sub-theme 2.1. Imbalance of Humors and Elements:** Traditional healers perceive illness as an effect of imbalance of three humors and five elements. Imbalance can be caused by various factors, including age, habits, food, lifestyle, and environment. When the balance or presence of these energies or elements is disrupted, it can lead to illness.

*“Wind imbalance in body can create headache, upset stomach, and pain below the waist. Asthma is also caused by wind imbalance. Wind imbalance is not even found through x-ray, but we can diagnose it through pulse”* (Age 72, Male)

**Sub theme 2.3. Digestive dysfunction:** Traditional healers emphasize that good food is essential for health, as it directly impacts liver function. They believe that any dysfunction in the digestive system, particularly involving the liver, leads to illness.

*“Illness is caused due to the deficiency of elements in our body and food habits. These days people do not have a balanced diet. Untimely meals also cause illness like acidity and stomach ache. Fried food and tea consumed by many these days is the cause for gastric problems. Meals should be taken timely.*” (Aged 52, Female)

It was reported that changing food habits and introducing modern food items like cooking oils, packed wheat, and rice, has been the primary source of illness among the people of Kinnaur. The staple food of Kinnaur had been barley, buckwheat, and tartary buckwheat, including some local vegetables. However, with modernization, people, especially the younger generation, gave up eating these food items and started consuming packed food items. Since food is the source of life, and it supports various energies that run the body, any imbalance in the food intake will result in illness.

*“Our changing food is the reason for disease causation. Earlier in Kinnaur we had never heard of diseases like BP, TB, Cancer, diabetes but now it is present among many people. The food we are taking now is not balanced, which results in disease”* (Aged 67, Male)

### 6.4 Theme 3. diagnostic techniques

It is the process of identifying illness and determining a person's health condition based on the individual's signs and symptoms. Unlike biomedicine, diagnosis is performed manually without the help of any equipment. Diagnostic techniques used by the traditional healers of Kinnaur start with questioning, followed by pulse and visual examinations.

**Sub-theme 3.1. Questioning:** Under the questioning method, the healer inquires about the patient's perception of his/her health condition. Instead, the conversation focuses on the symptoms of ill health, dietary patterns, and habits. The healer also examines the personal aspects of the individual seeking treatment, along with their social and cultural environment. The patient's case history offers valuable information to the healer. The diagnostic process not only seeks answers to the question of how the illness originated, but what caused the illness, and why it has affected this particular person.

“What happened, how, when, where it happened is asked of the patient and it is very important for the diagnosis” (Aged 56, Male)

The conversation between the two is generally done in the local dialect, which helps in understanding the problem and provides a basis for appropriate treatment. Whenever a patient comes for treatment, they are usually accompanied by family members or friends. Family members provide useful information about the local environment, the patient's behaviour, food intake, habits, and past medical history.

**Sub theme 3.2. Pulse examination:** Pulse (*nabz*) examination is the most important diagnostic technique used as it gives an insight into the patient's physiological condition. It accounts for the individual's three humors, i.e., *Vata, Pitta,* and *Kapha* (wind, bile, and phlegm), and imbalances in each organ specifying illness. The technique of pulse examination incorporates critical subtle elements, such as the best time of day to measure a pulse and the correct method of placing fingers when pursuing the beat. Pulse examination is generally carried out early in the morning when the patient has an unfilled stomach, as it gives the best idea about the patient's health. The efficacy of treatment depends upon the pulse examination, and it is also one of the measures to decide the completion of the training process of a healer.

**Sub theme 3.3. Examination:** It includes physical and urine examination. A physical examination of the body's shape, skin colour, tongue colour, and texture is conducted first. Any external injury, or infectious part of the body is examined and in cases of internal injuries, the examination is conducted by touch. Urine analysis is done by analyzing significant aspects of urine, like colour, odour, and formation. The first urine of the day is preferred for testing in a urine test.

Once the diagnosis and overall body assessment are completed, the specific illness is identified, and treatment begins.

### 6.5 Theme 4. treatment methods

Treatment is a gradual process involving three significant steps which are a) dietary and behavioural changes, b) oral medicines and ointments, and c) surgery, and other external therapies. This chronology helps in the appropriate treatment of the individual.

**Sub-theme 4.1. Dietary and Behavioural Changes:** The healer suggests post-diagnosis dietary changes in accordance with the patient's social, psychological, and physiological condition, keeping in view the ecological conditions prevalent in the region. In addition to dietary regulations, the timing of meals is also specified.

“*Timing of the meals should be fixed; taking meals at irregular intervals increases the risk of illness*” (Aged 76, Male)

Healers suggested that food intake timing plays a vital role in maintaining good health, as it helps maintain balance. Eating meals at odd times and irregularly affects an individual's health. Food taken at proper intervals provides good nutrition and boosts the body's immunity. Home-grown and locally produced food is strongly recommended to remain healthy.

“*Home grown food items which are chemical and fertilizer free should be taken as it never causes any disease. Food other than prepared in the house is strictly prohibited*” (Aged 59, Male)

“Balance diet” is key to a healthy life, and this is the fundamental treatment process by all the traditional healers of Kinnaur. The types of food recommended are *Sattu* (Barley), milk, curd, millet, and local cereals. The treatment also involves prohibiting certain food items, which generally include fried food and a high-fat diet. Along with diet recommendations, behavioural or lifestyle changes are also recommended. Alcoholic drinks, smoking, and other unhealthy habits are strictly prohibited. Appropriate sleeping habits, such as sound and timely sleep, are also strongly recommended. Hygiene and physical activity are motivated among the patient depending upon the age of the patient.

“*Patient has to sacrifice a lot, it is his/her choice whether he/she can follow the instructions or not but to get cured these are mandatory*” (Aged 65, Male)

**Sub-theme 4.2. Oral medicines, Oil and Ointments:** The most common mode of treatment is the prescription of herbal powder and pills. Local herbs are the major raw material for oral medicines and ointments. A combination of 2–30 herbs is used to create medicine for various illnesses. Traditional healers also recommend oil massages, which enhance blood circulation and aid in the healing process. These treatments are generally suggested for injuries related to muscles, tendons, ligaments, broken bones, migraines, arthritis, and more. A variety of plants in Kinnaur contain essential oils, which are extracted using indigenous techniques. Ointments in the form of paste are suggested for external as well as internal injuries.

**Sub-theme 4.3. Cupping and Bloodletting:** This is part of surgical treatment, and it involves the creation of a vacuum around the painful part of the body using cups made of metal. Before placing cups on the affected part, they are warmed by placing a flammable substance inside the cup, which removes the air inside and creates a vacuum. When placed on the skin, it creates a suction of blood on that spot, and blood is collected in the skin under the cup. This causes the skin under the cup to rise and redden as blood vessels expand. Cups are generally placed for five to 10 min until the skin under the cup becomes light red. The healer then removes the cup and uses a blade to cut at the point where blood has accumulated. This part of the technique is called bloodletting. The stagnant or impure blood is removed from the body, curing the pain. Earlier, only metal cups were used, made of gold, copper, and silver, but nowadays some healers use plastic and glass cups for treatment. This treatment is used for curing arthritis, muscular pain, and gastrointestinal problems.

**Sub theme 4.4. Moxa/Moxibustion:** ‘*Moxa*’ means applying heat directly on the affected part of the body, the basic principle is stimulating energy circulation in the body using heat which further cures the illness. ‘Moxibustion’ involves the use of herbs, fire, and instruments made of metal and wood. The conical shape instrument is filled with herb on the hollow side and set on fire, after setting the herb on fire the cone is flipped downward in a position so that the burning herb is in direct contact with the skin (without causing any damage to it) at the point of injury or pain on the body. It is used for treating arthritis, skin diseases, and blood-related disorders. Nowadays, these surgical methods are rarely used by healers because of the complicated techniques, and patients refrain from using them.

**Sub-theme 4.5. External therapies:** These are also known as soft surgery treatments, wherein the patient is advised to take a bath in warm water containing extracts of herbs or raw herbs. These are followed in case of skin diseases and muscular pain. A bathing tub is filled with herbs, usually in the form of extracts or leaves, which are immersed in water for more than half an hour, and then the patient is asked to take a dip for more than 20 min. In some cases, this is repeated for a week or more. At times, the patient is advised to bathe in natural hot water geysers containing natural minerals.

### 6.6 Theme 5. training and learning process

Traditional medicine healers are trained using a persistent schedule and substantial empirical research conducted under the guidance of a professional healer with vast experience in health care.

**Sub theme 5.1. Transmission of Knowledge:** The predominant form of transmission of medical knowledge happens through family lineage. Traditional healers have attained indigenous knowledge from their families, as their fathers and grandfathers are well-known healers of the area. This transmission of knowledge generally happens orally from one generation to the next. Commonly reported methods for transmission of knowledge include oral instructions, participant observation by the learner, and demonstrations by the provider. The learning process (diagnostic techniques and treatment) generally takes 8–10 years. Practical training forms a significant part of the transmission process, entailing vast field research in forest areas. Since most herbal plants used to make medicine are located at high altitudes, most of the time in the early years of training is spent in the forest gathering comprehensive knowledge about the flora and fauna. The learner should have appropriate knowledge of the region's local culture, social organization, and ecology. The training is divided according to the seasons, during summers, the collection of herbs and outdoor activities are conducted, whereas, due to heavy snowfall during winters, the theoretical concepts of the medicine system and preparation of medicine are taught.

**Sub-theme 5.2. Learning preparation of medicine:** Indigenous techniques of medicine preparation are imparted practically under the supervision of the traditional healer, and the techniques are observed when the healer prepares the medicine. This whole process of knowledge flow occurs orally, although in some cases it was observed that certain Buddhist healers refer to the Tibetan medicine literature known as “*Pothi*” in the local language. After 3–4 years, the apprentices always accompany the healer during diagnosis and treatment of patients, thus providing them with first-hand experience in healing techniques. Finally, the trainer assesses the apprentice and announces the completion of the training, after which the apprentice begins practicing independently in the healing profession. During the full course, the apprentice also discharged his duties as a member of his family and community by participating in everyday work and community festivals and fairs.

### 6.7 Theme 6. Production of medicine

The healers learn different steps, from collection of herbs to preparation of herbal medicine, during the training process. Traditional healers believe that the efficacy of traditional medicine purely depends on these steps.

**Sub theme 6.1 Identification and Collection of the plant:** Identifying the right plant among hundreds of wild-grown species requires years of learning and experience. Knowledge gathered from field visits and training with experienced healers is essential for recognizing plants based on the diseases they can cure. The geographical location and altitude of District Kinnaur is favorable for the growth of herbal plants. There are endless varieties of herbal plants available in different regions of the district; one can find herbs even in the vicinity of populated villages. The hill tops are home to herbal plants with some of the world's rarest herbs growing in the wild. Gathering herbal plants for pharmacopeia involves basic principles that are important for the efficacy of medicine. Firstly, the healer should know which part is to be used (stem, leaves, root, flower, etc.); secondly, the method of collecting herbs should be proper so that the species is not destroyed and continues to grow in the future. The growth of herbal plants depends upon the year's season; therefore, the healers need to harvest herbs during the growing season of the plant.

“*herbal plants are very rare things, we have to identify first, it is found in very difficult places, we harvest it in a way which helps it grow in the next season, before collecting root we have to see that plant has flowers and seed which will help it regrow next time, leaves and stems are extracted carefully so that the plant is not destroyed, this is nature’s gift for us and we have to take care of it. We learn all this from our teacher during field visits*” (Aged 76, Male)

The above narrative from the field explains the importance of indigenous methods of herb collection, which are also ecologically significant. For the collection of herbs, all healers reported that shepherds have very good knowledge of herbs and are well versed with the location of every herb grown nearby. A few elderly healers revealed that they used to visit Tibetan region in China to collect and trade herbs as it was legally allowed earlier. In addition, these visits also included exchanging traditional medicinal knowledge and participating in religious ceremonies. Due to the ecological similarities of both regions, some of the medicinal practices are similar, providing an opportunity for the exchange of traditional knowledge and discussing new possibilities to tackle diseases and illnesses. In the last decade, this exchange has been stopped as no one is allowed to travel across the Indo-China border.

**Sub-theme 6.2. Preparation Methods:** Medicine preparation is a stepwise process that involves indigenous techniques and traditional tools. After collecting different herbs, these are washed and dried (shade dried), and then the herbs are weighed with the help of a weighing machine (usually made of copper). The next step is to crush the herbs in a ‘mortar-pestle’ (*hamam dasta*), usually stone or metal. The stone grinder is also used to make fine herbal powder if required. This powder is filtered with the help of a sieve and packed in bottles or other containers. Traditional healers use stone tools to prepare herbal paste and ointment.

*“The methods of drying and crushing are different. Herbs should not be dried in the sun as it removes the herb's medicinal properties. It has to be dried in the form of a garland. Medicine prepared with the hand can cure any illness. It cures illness from its roots*” (Aged 52, Female)

Methods such as Hydro-distillation, oil mills, and decoction are used for oil preparation. Oil mills are one of the most traditional methods used in India for extracting oil. In Kinnaur, traditional healers use stone tools to produce oil, although it is a very time-consuming. However, these healers still rely on indigenous techniques that have been culturally incorporated through generations. These healers refrain from using any modern technology to make medicine. Traditional healers of Kinnaur strongly condemn the use of electricity in any of the preparation processes.

*“I don’t use electricity as it heats up the herbs resulting in low efficacy that is why I use stone tools for grinding”* (Aged 55, Male)

## 7 Discussion

The traditional medicine system of Kinnaur is an age-old setup without any organizational setup, based on the use of herbal plants found in the vicinity. The traditional healers of Kinnaur are an integral part of the societal fabric, universally recognized by the community members, and are expected to fulfill healthcare duties along with other diverse responsibilities, encompassing religious and cultural obligations. Sharing common culture and beliefs facilitates a nuanced comprehension of the healthcare needs of the community members. Traditional medicine practices may vary with country and region as they are influenced by culture, history, personal attitude, and philosophy [13]. Similar to the traditional medicine system of Kinnaur, traditional Chinese medicine is also one of the oldest medicine systems developed 2000 years ago, also known as the Yellow Emperor's medicine classic [14]. Traditional Healers have widespread popularity in most of the LMIC's (Low- and middle-income countries). They continue to play an important role in the healthcare systems largely due to shared cultural values and beliefs [7,8].

Holistic concepts of health and illness among the traditional healers of Kinnaur revolve around the balance of three humors and five elements of the human body. Similar concepts of the three humors are popular among the Siddha and Ayurveda systems of medicine [33] and also fundamental concepts of SowaRigpa [15], stating that each humor has a different function [16]. Similar to the present concept of health, Hippocrates believed in the balance of four humors, which are sanguine (blood), choleric (black bile from the liver), phlegmatic (phlegm), and melancholic (yellow bile from the kidneys) [17]. Concepts of holistic treatment aiming at balancing the physical body, cultural, Soul, and spiritual realms also exist among African healers [18].

The dynamics of knowledge transfer, and preparation of herbal drugs have been documented in other indigenous and tribal systems as well. Langwick [19], in her examination of healing practices that Tanzanians use, highlights that traditional healers derive their legitimacy both culturally and in terms of embodied knowledge. The same applies to the practice of healers among Kinnaura, who use experiential learning, religious knowledge, and individualized diagnostic techniques in healing diseases.

Traditional healers in Kinnaur believe that treatment involves helping individuals restore balance among the various forces and elements within their bodies. They emphasize the significance of a balanced diet for maintaining overall health, as it plays a crucial role in both preventive and curative care. According to these healers, health is a result of the harmony between the mind and body, and a balanced diet is essential for achieving this equilibrium. Similar concepts about the effects of behavioural and psychosocial stress on the physiology and health of an individual have been documented by Baum and Posluszny [20], Salovey et al. [21]. Traditional healers also believe that illness is related to digestive dysfunction (lever) and accounts for changing food habits of the Kinnaura as the significant reason for illnesses like Blood pressure (B.P), and Diabetes.

The diagnostic technique applied by the traditional healers of Kinnaur is holistic in approach as it includes all aspects of an individual (Physiological, Psychological, and Socio-cultural). It starts with questioning, which provides the healer with an idea of the lifestyle of the patient, his/her dietary habits, and socio-cultural environment. Questioning is a casual conversation between the healer and patient, which helps both the healer (in assessing the patient's condition) and the patient (believing in the treatment process). Traditional healers of Ethiopia, known as Medhanit Awakis, also use questioning methods for diagnosis and sometimes subscribe to medicine based on patient descriptions [22]. In Kinnaur, questioning is the first step of the diagnosis, followed by a pulse examination. Pulse examination is also reported among the Siddha and Unani medicine systems and possibly followed by Ayurveda in the 11th century after coming in contact with these two systems. A similar practice was also reported among the folk healers of Jaunpur (Uttar Pradesh), who believed that the pulse's shape, size, and motion help determine health problems [23]. *Jariyara* (traditional healer), who belongs to the Jaunsari Tribe of the Central Himalayan region, also believes that the pulse reveals everything about the illness [24]. Following pulse examination, traditional Kinnaur healers conduct visual examinations, including observing the shape and colour of skin or particular organs, body heat, and the affected part of the body. Sometimes, a urine sample is also visually examined and diagnosed; the first urine of the day is preferred. Traditional healers of the Lohit District of Arunachal Pradesh known as *Chau ya* have also been reported to diagnose urine samples for the treatment of diabetes mellitus [25]. After implementing these diagnostic techniques, illness is specified and treatment begins, which involves dietary and behavioural changes, herbal medicine, and external therapies. Surgical methods used by healers are Cupping, Bloodletting, and Moxibustion, which are performed using indigenous tools made of wood and metal. Moxibustion is an important treatment technique used widely in the traditional Chinese medicine system ([26]; Du et al., 2007; [34]). The traditional healers of Kinnaur prepare medicine using indigenous techniques like grinding and decoction. It is observed that the healers do not use any electric machine for making medicine as they believe that electricity heats the raw herbs or the components of herbs may react with the metal (grinding machine), resulting in decreased efficacy of the medicine. No research has been conducted to find the adverse effects of electricity on the preparation of herbal medicine. Indigenous preparation methods, from the collection of herbs to making medicine, maintain the efficacy of medicine, and traditional healers of Kinnaur strongly believe that if changes are made in these methods, they will not yield positive results. The significance of indigenous methods of medicine making was illustrated by Tu Youyou, which led to the extraction of artemisinin (anti-malaria) from the Chinese herb Qinghaosu, for which she was conferred the Nobel Prize in 2015 in the field of Medicine [27].

The transmission of indigenous knowledge happens through family lineage, and in some cases, friends and fellow traditional healers. Lozada [28] also documented a similar source of transmission of traditional medicine knowledge among the rural community of Northwestern Patagonia, Argentina, where most people become healers after training under their grandparents or parents. (Mwaka et al., 2023). Common methods reported for learning by the healers of Kinnaur (informal) included oral instruction, demonstration, and participation. Similarly, Limpopo Province of South Africa healers also learn traditional knowledge through inheritance from their parents or grandparents [29,30]. Socio-cultural aspects are integral to traditional medicine; trainees should learn about them while learning medicine. Many researchers have demonstrated that cultural beliefs shape the understanding of health and illness, and affect treatment seeking behaviour (Klienmen et al., 1978; [31]). In Kinnaur, the conversation between the patient and provider regulates a two-way flow of knowledge, and the patient never feels isolated from the diagnostic and treatment process. The accomplishment of compliance between the two helps provide appropriate healthcare service, and similar findings have been documented by various researchers [32], who stressed the role of the patient and provider relationship in the treatment process. Belief in traditional medicine is one of the reasons why some people still use this age-old tradition, as it provides permanent solutions to illness and mental satisfaction during the treatment process. It is observed that the felt needs of the patient are understood by the local healers more comprehensively than in modern health care, where the patient (especially the elderly) is at a loss to understand the inference drawn by the doctor.

## 8 Conclusion

The traditional medicine system of Kinnaur is a culturally defined healthcare system developed based on indigenous beliefs and empirical knowledge gathered by community members or groups. Traditional healers of Kinnaur have a holistic view of health and illness, dealing with all aspects of the individual (physiological, psychological, and socio-cultural). All the years gone by this knowledge was handed over to the next generations orally (verbally), and it prevailed and flourished until now. If this knowledge is not documented or transferred to the next generation, the age-old tradition may become extinct. With the loss of this system, a lot of preventive, promotive, and curative knowledge will fade away, and the lack of proper implementation of health policies on the traditional system of medicine increases these chances. It is the need of the hour that efforts should be made to save the traditional system of medicine in the presently changing society so that such rich knowledge is preserved and used for the betterment of humans. Participatory research will help in understanding traditional medicine within the cultural context of a tribe/community and will help incorporate indigenous knowledge into the health programs. After independence, we have overlooked these systems, labeling them as non-scientific. This neglect has contributed to their decline. It is essential to conduct in-depth interdisciplinary studies on the natural resources in the area to fully understand their value. Many studies have been conducted on the classification of plant species, but there are very few studies documenting the active components of herbal plants. In-depth interdisciplinary studies on the area's natural resources should be undertaken to understand active compounds of herbal plants used by traditional healers. This research can help validate traditional medicine through rigorous biomedical investigation. Healthcare policies should prioritize the integration of socio-cultural factors to deliver holistic healthcare services. By understanding these socio-cultural patterns, policymakers can create strategies that preserve traditional medical knowledge. This understanding will also help healthcare providers become more aware of and sensitive to the beliefs and practices of their patients, allowing for a more comprehensive approach to patient care.

## Author contributions

Abhishek Sharma: Conceptualization, Methodology/Study design, Validation, Formal analysis, Investigation, Resources, Data curation, Writing – original draft, Writing – review and editing, Visualization, Supervision, Project administration.

Qutsia Tabasum: Conceptualization, Methodology/Study design, Validation, Formal analysis, Investigation, Resources, Data curation, Writing – original draft, Writing – review and editing.

## Declaration of generative AI in scientific writing

The authors declare that no generative artificial intelligence (AI) tools were used in the writing, editing, data analysis, or preparation of this manuscript.

## Funding sources

This research was independently conducted without external funding support.

## Conflict of interest

The authors declare that they have no known competing financial interests or personal relationships that could have appeared to influence the work reported in this paper.
